# Molecular and evolutionary characteristics of the fraction of human alpha satellite DNA associated with CENP-A at the centromeres of chromosomes 1, 5, 19, and 21

**DOI:** 10.1186/1471-2164-11-195

**Published:** 2010-03-23

**Authors:** Nathalie Pironon, Jacques Puechberty, Gérard Roizès

**Affiliations:** 1 Institut de Génétique Humaine, UPR 1142, CNRS, 141 Rue de la Cardonille, 34396 Montpellier Cedex 5, France

## Abstract

**Background:**

The mode of evolution of the highly homogeneous Higher-Order-Repeat-containing alpha satellite arrays is still subject to discussion. This is also true of the CENP-A associated repeats where the centromere is formed.

**Results:**

In this paper, we show that the molecular mechanisms by which these arrays evolve are identical in multiple chromosomes: i) accumulation of crossovers that homogenise and expand the arrays into different domains and subdomains that are mostly unshared between homologues and ii) sporadic mutations and conversion events that simultaneously differentiate them from one another. Individual arrays are affected by these mechanisms to different extents that presumably increase with time. Repeats associated with CENP-A, where the centromere is formed, are subjected to the same evolutionary mechanisms, but constitute minor subsets that exhibit subtle sequence differences from those of the bulk repeats. While the DNA sequence *per se *is not essential for centromere localisation along an array, it appears that certain sequences can be selected against. On chromosomes 1 and 19, which are more affected by the above evolutionary mechanisms than are chromosomes 21 and 5, CENP-A associated repeats were also recovered from a second homogeneous array present on each chromosome. This could be a way for chromosomes to sustain mitosis and meiosis when the normal centromere locus is ineluctably undermined by the above mechanisms.

**Conclusion:**

We discuss, in light of these observations, possible scenarios for the normal evolutionary fates of human centromeric regions.

## Background

Although human alpha satellite DNA sequences have been studied for decades, a number of their structural and evolutionary characteristics remain obscure. It is generally accepted that sequences constituting highly homogeneous arrays, including those within which the active centromere is formed, evolve in a concerted way [1]. In view of this concerted evolution, many authors have supposed that the repeats are homogenised with high efficiency, both intra-chromosomally and between homologues. At the same time, it has been shown that meiotic recombination is highly suppressed in the centromeric chromosomal regions [2-5]. Indeed, it was recently shown that homologues can bear subsets of Higher Order Repeats (HORs) that differ by a number of Diagnostic Variant Nucleotides (DVNs), indicating that exchanges between the homologues are at most highly limited [6].

Multiple molecular mechanisms are thought to underlie concerted evolution, principally unequal crossing over and gene conversion. Two recent papers have discussed this in detail: Schindelhauer and Schwarz [7] proposed that conversion, as opposed to unequal crossing-over, was the dominant mechanism behind the homogenisation of the HORs on chromosome X. Roizès [6], on the other hand, using the examples of chromosomes 17, 13, and 21, mainly considered unequal crossing over and suggested that conversion rather introduces divergence between the repeats of homogeneous arrays. It is difficult, however, to reconstruct the course of homogenisation of alpha satellite repeats in the absence of their map positions.

The fraction of the repeats within the homogeneous alphoid array at which CENP-A is recruited with other proteins [8] to form the centromere has never been analysed in detail. In particular, it is not known whether these repeats differ from the other repeats in the array. Interestingly, it has been recently shown that the repeats associated with the active centromeric chromatin of *Arabidopsis thaliana *and *Zea mays *are hypomethylated relative to the same repeats within the flanking pericentromeric chromatin [9].

In this paper, we have further analysed the highly homogeneous arrays of a number of chromosome homologues (1, 3, 5, 19, and 21). Our analysis essentially confirms the initial results of Roizès [6], although the data are somewhat more complex and diverse than originally proposed. The D1Z5 locus appears to be archetypical of the mode of evolution of these sequences. The fraction of the repeats associated with CENP-A was also analysed (chromosomes 1, 5, 17, 19 and 21); this analysis revealed that, while the CENP-A associated repeats evolve by the same molecular mechanisms as the other repeats, they constitute subsets that exhibit different combinations of DVNs and thus distinct domains and subdomains within the overall centromeric array. Negative selection seems to be acting during the homogenisation/amplification runs which drive them. On chromosomes 1 and 19, CENP-A associated alphoid repeats were recovered from two different and unrelated homogeneous arrays. These results are discussed in light of possible mechanisms for the formation, evolution, and loss of centromeres.

## Results

### Analysis of a long stretch of HORs belonging to locus D1Z5

Although there is a large amount of alpha satellite DNA sequence data in genomic databases, it was difficult to find sufficiently long, uninterrupted stretches of such DNA among the numerous BACs that had been partially or totally sequenced. An examination of the maps of all the human chromosomes available on the web http://www.ensembl.org/Homo_sapiens/mapview?chr failed to yield any more information in this regard, as most arm junctions within the alphoid contigs reported therein lacked highly homogeneous alpha satellite HORs. Examining the published sequences of entire human chromosomes also mostly failed to offer any additional useful information. The only two exceptions concerned chromosomes X [10] and 8 [11]. The X and 8 array junctions contain 21 kb and 44 kb, respectively, and 2 × 18 kb of highly homogeneous DXZ1 and D8Z2 HOR sequences on the p and q arms. A ClustalW alignment (not shown) of these repeats showed that the homogenisation processes acting on the two edges of DXZ1 and D8Z2 are independent, indicating that the two alpha satellite DNA sequences do not exchange with each other at a distance.

We also identified one BAC that contains a long insert of entirely assembled alpha satellite DNA. It originates from chromosome 1 and had been sequenced and assembled in NCBI: BX248407 (gi: 45535739). The assembly was confirmed by restriction digestion. It contains 141,084 bp of contiguous alpha satellite DNA. *In silico *restriction analysis revealed that its central part consists of 1866 bp-long HORs (11 times the basic 171-bp repeats), whilst the two sides contain more divergent DNA sequences. It thus corresponds to locus D1Z5, which had been previously characterised as generating 1.9 kb DNA fragments upon restriction by Hind III and as covering 100-300 kb [12].

**Figure 1:**
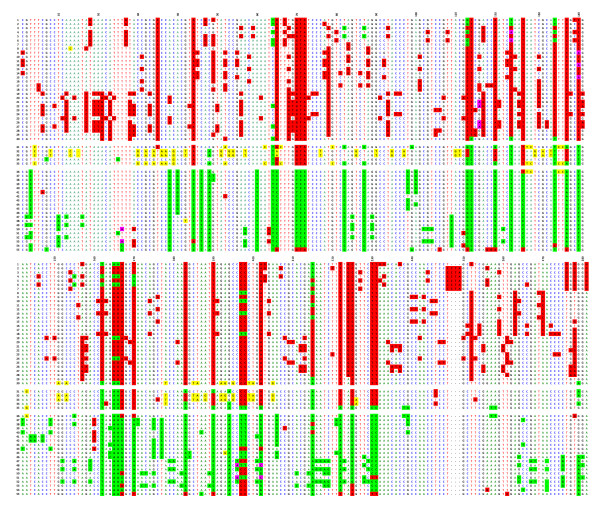
**ClustalW alignment of the 55 complete HORs of BAC BX248407**. Alignment was obtained using the ClustalW program (35). The HORs are ordered from 1 to 55 as they appear in the published DNA sequence. DVN positions are indicated above the alignment from 1 to 141 and from 142 to 281. The remaining positions are not shown, as they are identical in all repeats; the same holds true for the other figures. DVNs shared by subset 1 (repeats 1-30) are shaded in red, those shared by subset 2 (repeats 36-55) are shaded in green, and those specific for subset 3 (repeats 31-35) are shaded in yellow. When DVNs are of two types at the same nucleotide position, purple shading is also used. Repeats 32 and 33 have presumably been generated by an unequal crossover event. Shared nucleotides are coloured in green for A, blue for C, red for T, black for G and "-" is used for deletions.

A ClustalW alignment of the 55 repeats of its central section is shown in Figure 1. Of the 1866 positions within the entire HOR, 281 correspond to Diagnostic Variant Nucleotides (DVNs), as defined in Roizès [6], as they are shared between at least 2 of the 55 copies. Although their distribution appears to be rather complex, it is striking to note that repeats 1 to 30 (subset 1) share common DVNs, as do copies 36 to 55 (subset 2), but that there is virtually no overlap between these two subsets. An intermediate subset 3 (copies 31 to 35) is present between subsets 1 and 2, with the two adjacent copies 32 and 33 being almost entirely identical and largely different from those of subsets 1 and 2. Sharing of DVNs between subsets 1 and 2 is quite limited, indicating that their respective repeats exchange almost entirely within each subset. Presumably, subset 3 constitutes an almost impassable barrier between subsets 1 and 2, perhaps by rendering the repeats belonging to the flanking subsets too distant from each other.

The most likely explanation for these observations is that the copies of each subset have been homogenised and amplified by an accumulation of crossovers, creating homogeneous domains as postulated by Roizès [6]. Once such a domain has been formed, exchanges continue in the same mode, with adjacent repeats engaging in unequal crossing over and thereby creating new subdomains, as is visible in both subsets 1 and 2. Unequal crossing over is often accompanied by conversion events, or can alternatively be aborted and reduced to short conversion events. It is easy to infer from the respective sequences of the subsets that a complete unequal crossover together with conversion has generated this overall structure. Further, one can note that there have been no more exchanges between subsets 1 and 2 since this original event occurred.

We can therefore conclude that the basic mechanism underlying the establishment of the structure encountered in this portion of the pericentromeric region of chromosome 1 (from which the BAC was isolated) basically corresponds to that described in Roizès [6]: an accumulation of unequal crossovers and the resulting creation of new alphoid domains by amplification and/or homogenisation. Mitotic crossing-over events can only occur between repeats that are in close linear vicinity and are therefore almost identical in sequence. The domains are rather small and are in permanent evolution, both by the accumulation of unequal crossovers and by superimposed multilateral conversion events. This also supports our suggestion that conversion introduces divergence rather than homogeneity.

**Figure 2:**
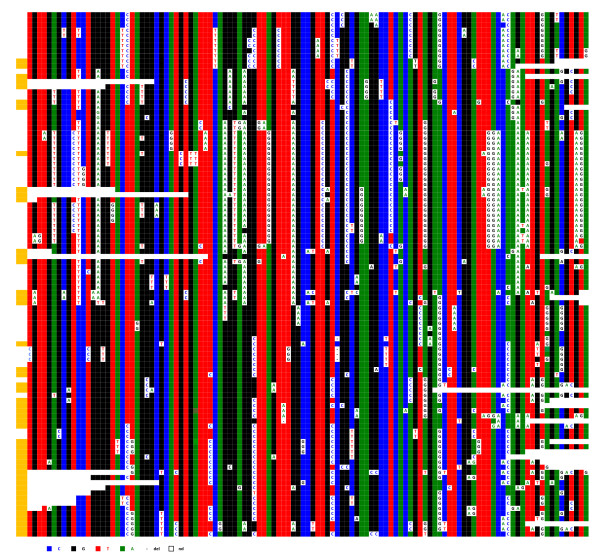
**The common portion of the repeats of both BX248407 and the corresponding locus of the GM 13139 homologue were ClustalW aligned simultaneously**. In the first column, those from GM 13139 are shaded in yellow. Overall, this figure shows that the repeats of the two homologues are indeed quite different in their DVN distribution and combination into haplotypes.

We next compared this first locus (D1Z5, from BX248407) to its orthologue on a chromosome 1 homologue from the hybrid cell line GM 13139. We proceeded using the approach described in Roizès [6]: PCR and sequencing of a number of cloned repeats. However, we were only able to analyse a portion (approximately 730 bp) of the 1866 bp-long HOR from GM 13139, and we did not know the relative positions of the repeats along the corresponding alphoid DNA block. We first ClustalW aligned the BX248407 repeats corresponding to this reduced HOR portion from GM 13139. In this case as well, the repeats were distributed in the same three distinct, non-overlapping subsets described above, although the copy order within each subset was not entirely the same. This allowed us to compare the copies present in the two homologues in a simultaneous ClustalW alignment (Figure 2). The GM 13139 repeats also exhibited short domains and subdomains comprised of copies bearing DVNs, with most of them, but not all, being in common with those of BX248407. Only a minority of the copies, however, share closely related haplotypes with those of BX248407, with most being largely unrelated. As above, this indicates that the two lineages bearing the two homologues (the one from which BAC BX24807 was constructed and GM 13139) are engaged in continuous and independent homogenisation/amplification (by accumulation of unequal crossovers) and diversification (by conversion) processes.

### Is the locus corresponding to BAC BX248407 an archetype of the evolutionary mechanisms operating within highly homogeneous alpha satellite arrays on all chromosomes?

As locus D1Z5 appears to be more complex than those reported in Roizès [6], we decided to analyse other alphoid sequences as well, specifically those from chromosomes 21 (D21Z1) and 3 [13], and those shared by chromosomes 1 (D1Z7), 5 (D5Z2) and 19 (D19Z3) [14].

**Figure 3:**
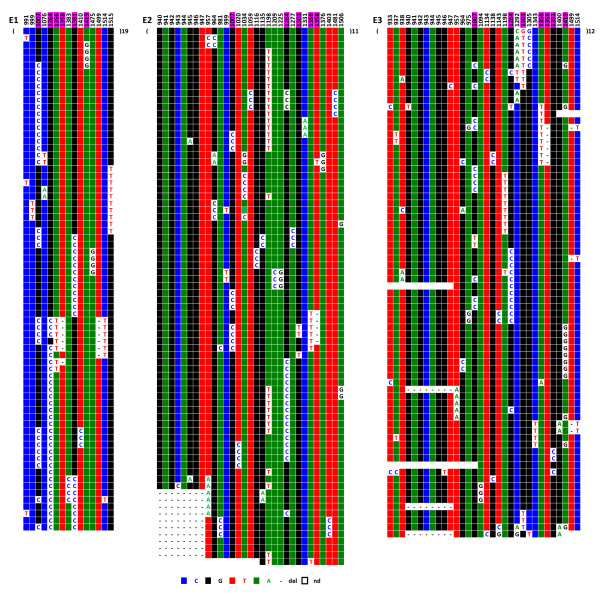
**ClustalW alignment of repeats from alphoid array D21Z1 of chromosome 21, E1-E3 (E4-E6 are shown in Additional file **1). The DVNs are shown in the upper line, with their positions along the 1866 bp long HOR. A minority of them are shared by the six homologues. 19 (E1), 11 (E2), 12 (E3) repeats exhibit no DVNs at all; they might, however, differ by a few sporadic mutations (less than 0.2% on average).

Ninety-six cloned repeats from six chromosomes 21, each isolated in a separate hybrid cell line, were sequenced, aligned, and compared to one another. The analysis was limited to a third of the chromosome 21 HOR (608 nucleotides out of the 1866 for the entire repeat length). The comparison confirmed that the repeats are much more homogeneous than those of the D1Z5 locus corresponding to BAC BX248407: the rate of sporadic mutations is very low (0.2% on average), and the number of DVNs is much lower than at the D1Z5 locus. It was also difficult to identify conversion events, which would have added to the sequence diversity of the locus. A high proportion of the repeats from the six chromosomes 21 share identical haplotypes (Figure 3 and Additional file 1), which is also indicative of the relative stability of this locus compared to D1Z5. Nevertheless, it appears that the DVNs are only partially shared between the six homologues, indicating that the homologues correspond to chromosome lineages that were separated long ago and are now evolving independently.

**Figure 4:**
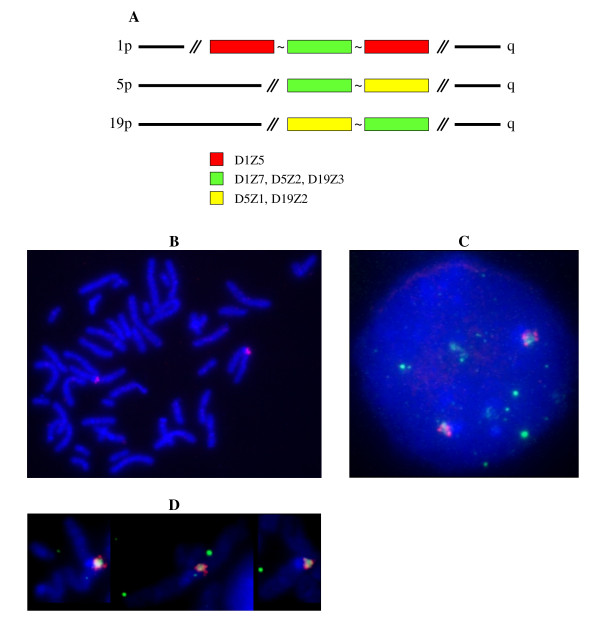
**A) Schematic representation of the centromeric regions of chromosomes 1, 5 and 19**. The three chromosomes share an alphoid array where the active centromere normally forms: D1Z7, D5Z2, and D19Z3 (15). As shown by Finelli et al (14), D1Z7 is embedded within D1Z5. Chromosomes 5 and 19 share a second alphoid array: D5Z1 and D19Z1. *In situ *hybridization with both pZ5.1 DNA (green signal) and BAC RP11-483B6 DNA (red signal) as probes on chromosomes in prometaphase (B, D) and nuclei (C).

Chromosomes 1, 5, and 19 all exhibit strong signals at their respective centromeres in FISH experiments using pZ5.1 as a probe, even at high stringency [14] (Figure 4B-D). The centromeric status of the corresponding loci (D1Z7, D5Z2, and D19Z3) has also been confirmed by the binding of CENP-C to these alphoid arrays [15]. When we used BX248407 DNA as a probe with Cot1 as a competitor, only chromosome 1 was labelled, confirming the arrangement of D1Z5 and D1Z7 shown in Figure 4A.

**Figure 5:**
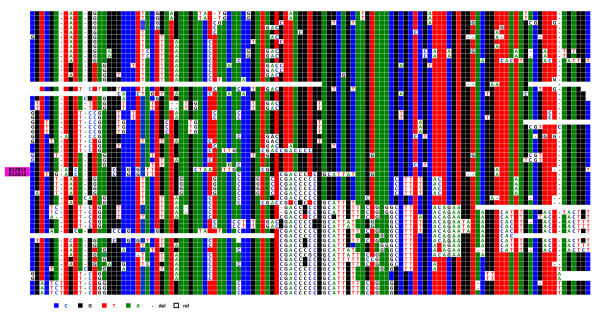
**ClustalW alignment of the sequenced repeats of D1Z7 from hybrid cell line GM 13139**. ClustalW alignment was performed with all sequenced repeats, including those in which a DVN is only shared by two repeats. Those DVN positions are not included here for more clarity.

An examination of the ClustalW alignments of the cloned repeats corresponding to these loci revealed that the three chromosomes exhibit an organisation similar to locus D1Z5, albeit with different degrees of resemblance. This is exemplified by chromosome 1 (Figure 5), where several homogenisation runs superimposed on one another are clearly visible, similar to BX248407. B12B12 and D12D12 share a portion of their respective sequences, which could have resulted from a conversion event occurring during an aborted crossover event between the two copies. Several multilateral conversion events are also easy to identify. Overall, 171 DVNs are detectable over the 652 nucleotides of the sequenced repeats, which is an even larger proportion than that observed at the D1Z5 locus. This, together with a proportion of sporadic variation of about 0.5%, reflects a high degree of exchange activity. The same holds true for chromosome 19, where again 0.5% of sporadic mutations were observed and which contains an even higher number of DVNs (227 over the 652 nucleotides of the sequenced repeats) (Additional file 2). The repeats of the two chromosomes are therefore engaged in a permanent turnover process based on an accumulation of crossover events, complete or aborted, associated with conversion events, as with the D1Z5 locus of chromosome 1.

**Figure 6:**
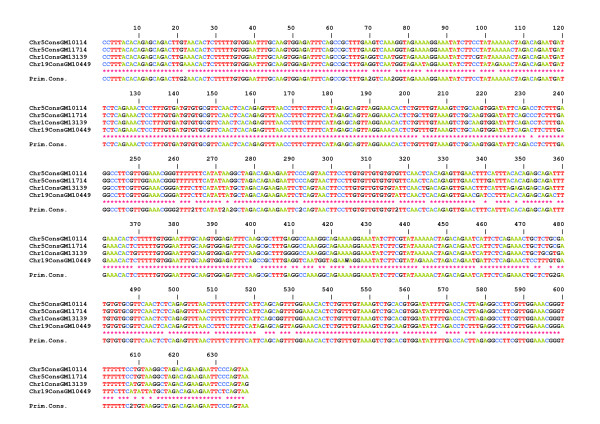
**Alignment of the consensus sequences obtained from those determined in this study from the alpha satellite of D1Z7, D5Z2 (for the two homologues of this study) and D19Z3**. Using FISH with oligonucleotides at positions where the consensus sequences were different, it was indeed possible to label specifically the chromosome with the specific consensus nucleotide (Toutirais, G, Witkowska, M, Piazza, A, Richard, F, Roizès, G and Escudé, C, submitted).

**Figure 7:**
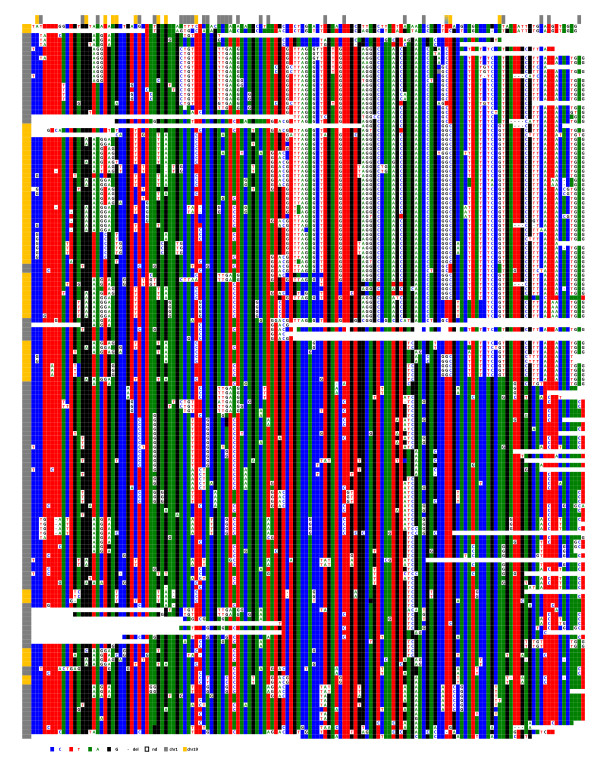
**Simultaneous ClustalW alignment of the repeats recovered from loci D1Z7 and D19Z3 from hybrid cell lines GM 13139 (shaded in grey) and GM 10449 (shaded in purple)**. The clustalW alignment was performed with all sequenced repeats, including those in which a DVN is only shared by two repeats. Those DVN positions are not included here for more clarity. Vertical arrows point to positions which have been specifically homogenised within one or the other chromosome.

In view of this permanent, ongoing process, it is easy to understand why, despite the almost identical consensus sequences of the HORs of chromosomes 1 and 19 (Figure 6), numerous positions have been homogenised specifically within each chromosome. This can be observed when the two sets of repeats are ClustalW analysed together, as they cluster separately (Figure 7).

**Figure 8:**
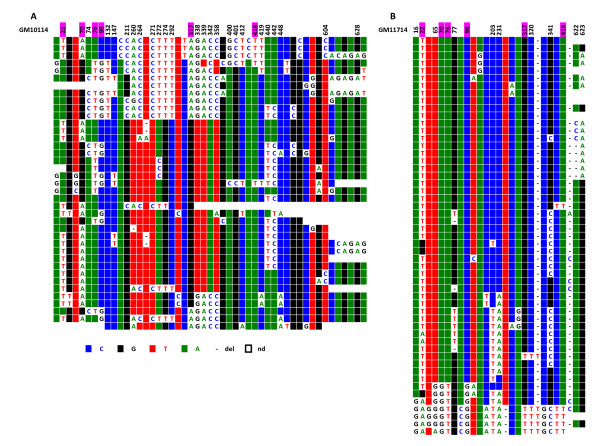
**ClustalW alignment of locus D5Z2 from the chromosome 5 of the two hybrid cell lines GM10114 (A) and GM 11714 (B)**. Above each set of sequences the nucleotide positions where a significant subset of repeats exhibit the same DVNs are indicated.

Repeats from two chromosome 5 homologues (Hybrid cell lines GM 10114 and GM 11714) were also compared. Interestingly, they looked intermediate in terms of their diversity between chromosome 21 on the one hand and chromosomes 1 and 19 on the other: the proportion of sporadic mutations was 0.3%, and the number of DVNs was much lower than in chromosomes 1 and 19 (74 and 44 for GM 10114 and GM 11714, respectively) (Figure 8). A large renewal of the DVNs has also occurred since the separation of the two lineages, indicative again of independent amplification/homogenisation runs.

Four chromosome 3 homologues were also examined using the same approach. By ClustalW alignment, it was possible to conclude that they are evolving according to the same rules as the other chromosomes analysed above (not shown).

### What are the characteristics of the alphoid DNA sequences associated with CENP-A within the centromere?

A small proportion of the repeats within a given homogeneous alphoid array are generally engaged in the functional centromere [16,17]. We decided to examine these particular repeats to determine if they exhibit sequence specificities and also to ask if they evolve as fast as the other repeats within the homogeneous array.

**Figure 9:**
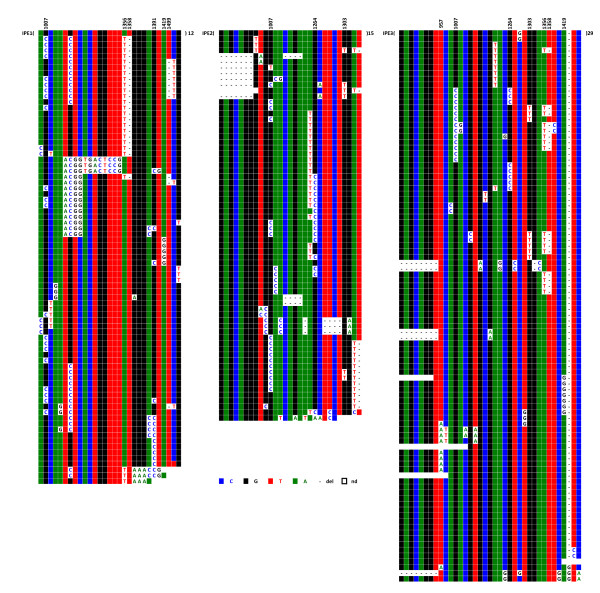
**ClustalW alignment of the IPE1-IPE3 (IPE4-IPE6 are shown in Additional file **3) **CENP-A associated repeats recovered by immunoprecipitation from chromosome 21**. The minority of DVNs that are shared between most of the six homologues are shown in the upper line, with their positions along the 1866 bp long HOR indicated. No DVNs at all were exhibited by 12 (IPE1), 15 (IPE2), or 29 (IPE3) repeats; they might, however, differ by a few sporadic mutations (less than 0.2% on average)

Six hybrid cell lines, each containing a single chromosome 21, were analysed. Centromere-associated repeats were recovered after immunoprecipitation of chromatin using a CENP-A specific antibody. Figure 9 shows the results of IPE1-IPE3 (IP for Immuno-Precipitated); results for IPE4-IPE6 are shown in Additional file 3. The analysis revealed that these CENP-A associated repeats also apparently evolve by the same molecular mechanisms as most of the repeats of D21Z1. One can, in addition, observe a clear difference between the CENP-A associated repeats (IPE1-6) and the bulk repeats (E_1_-E_6_) with respect to the distribution of DVNs and their combinations in a large majority of the repeats. However, perhaps due to their very low variability, a significant minority of the IPE_1-6 _repeats shared the same haplotypes as E_1-6_, so we could not firmly conclude that the subset of alphoid sequences involved in the formation of active centromeres is entirely different from the other repeats. Finally, in both cases, the homogeneity is lower than in E1-E6.

**Figure 10:**
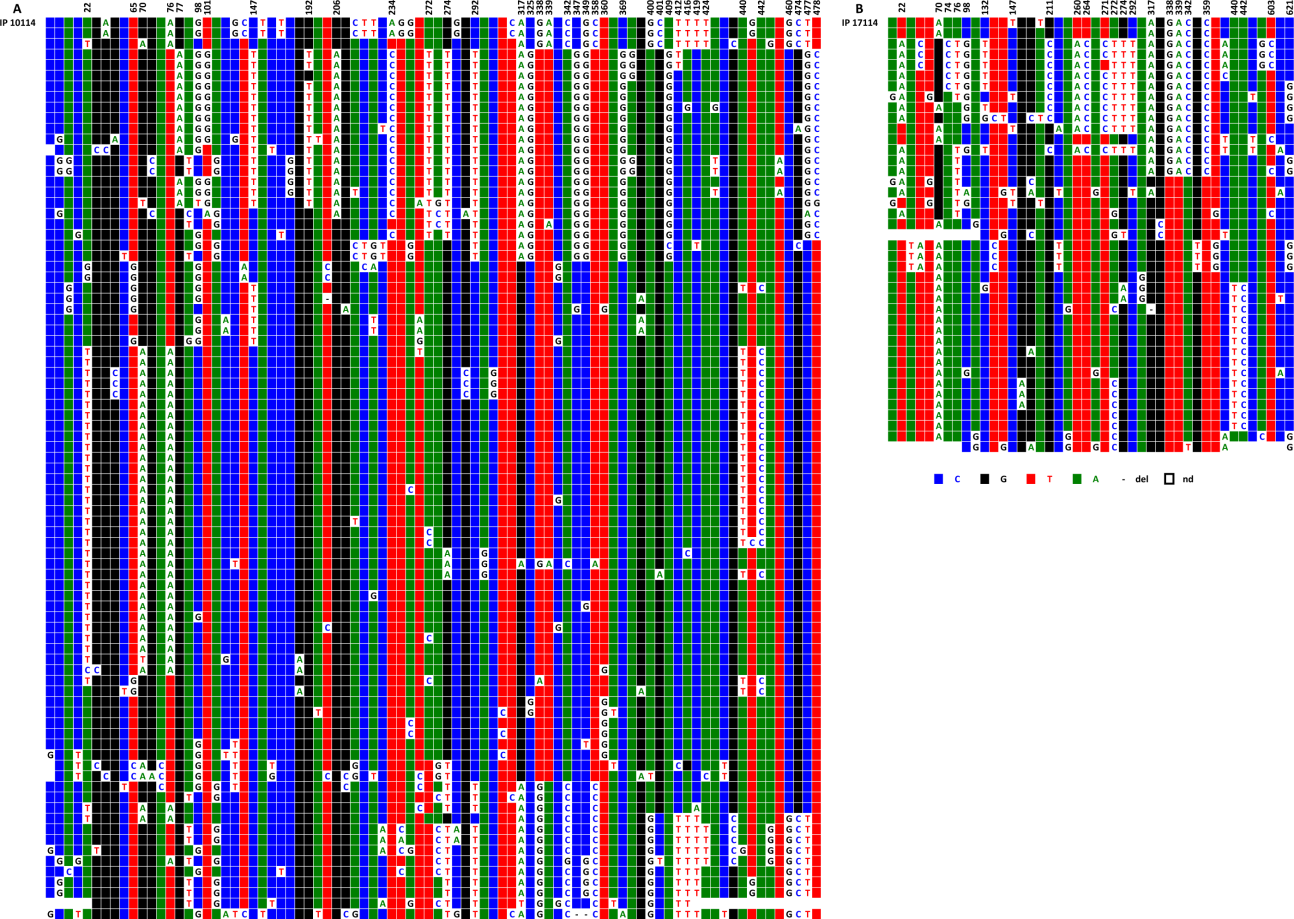
**ClustalW alignment of the IP10114 (A) and IP11714 (B) CENP-A associated repeats recovered by immunoprecipitation from the two chromosome 5 homologues**. Above each set of sequences the nucleotide positions where a significant subset of repeats exhibit the same DVNs are indicated.

When chromosomes 1, 5, and 19 were examined, it proved impossible to identify repeats exhibiting the same haplotypes in the two sets (centromeric and pericentromeric) within each chromosome, indicating that the repeats involved in the formation of the centromere only represent a very small percentage of the overall repeats; they do, however, follow the same type of evolution. Moreover, the respective DVN distributions within the centromeric and pericentromeric repeats were clearly different. Statistical analysis was not, however, performed on these sequences, as the size of the alphoid blocks was unknown and as it was impossible to determine whether the minority of repeats with the exact same sequence were independent or duplicate clones. This is illustrated by the two chromosome 5 homologues analysed in this study (Figure 10). The same holds true for chromosomes 1 and 19 (Additional files 4 and 5).

As the D1Z5 locus could represent an archetypical structure for the homogeneous alphoid arrays analysed here, we next asked whether it was possible to recover alphoid repeats after chromatin immunoprecipitation with anti-CENP-A. To do this, we PCR amplified the DNA repeats corresponding to D1Z5 from the same sample of GM 13139 that was used to obtain the repeats of D1Z7, where the centromere is known to form [15]. CENP-A associated repeats were indeed recovered, cloned, and sequenced. Their clustalW alignment revealed the same type of pattern as their bulk counterparts, with less complexity and, hence, more homogeneity. It is noteworthy that, as shown by FISH (Figure 4), D1Z5 is present within D1Z7 [14].

**Figure 11:**
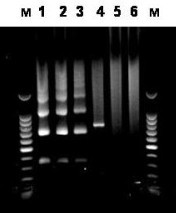
**PCR amplification of the DNA recovered by immunoprecipitation from chromatin with an anti-CENP-A antibody**. Samples were from Chr 19 (GM 10449): 1 and 4; Chr 5 (GM 10114): 2 and 5; and Chr 5 (GM11714): 3 and 6. Amplification was performed with oligoprimers specific for D19Z3 and D5Z2, shared with Chr 1 for both chromosomes: 1, 2 and 3. The locus common to chromosomes 5 and 19 only was amplified with the appropriate oligoprimers: 4, 5 and 6. M = 100 bp marker.

This observation prompted us to investigate whether the same was true for chromosomes 5 and 19, as they also share a second homogeneous alphoid array [12] (D5Z1 for chromosome 5, D19Z1 for chromosome 19). To do this we again used the same DNA samples obtained by ChIP that had been used to analyse the alphoid repeats specifically associated with D1Z7 (chromosome 19) and D5Z2 (chromosomes 5 from GM 10114 and GM 11714). A PCR amplification assay with primers specific for this array revealed CENP-A associated repeats for chromosome 19, but not for the two chromosomes 5 (Figure 11).

Two previously described [6] hybrid cell lines, each containing a single chromosome 17, were also analysed with respect to their centromere-associated repeats. GM 10321 exhibits HORs which are 16- and 13-mers of the basic 171 bp alphoid unit, whilst GM 10498 only has the 16-mer [6]. The CENP-A associated repeats were analysed by sequencing and ClustalW alignment (not shown). Again, the same properties were detected as described above.

**Figure 12:**
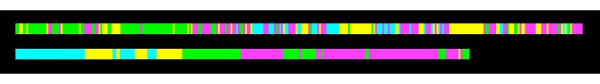
**Schematic representation of the simultaneous ClustalW alignments of the alphoid sequences obtained from D1Z7 (coloured in green), D5Z2 (yellow for GM10114 and blue for GM11714), and D19Z3 (purple)**. Top: CENP-A associated repeats; bottom: repeats from the bulk sequences. Each rectangle is strictly proportional to the number of repeats which were found clustered in the alignment.

Although there is no such indication in the literature [18], we tried to determine if a subtle sequence specificity of the alpha satellite repeats could contribute to the formation of nucleosomes using CENP-A as a substitute for histone H3. It was difficult, for this purpose, to compare alphoid sequences belonging to different chromosomes, as their sequence differences are generally relatively high. This was not true, however, for chromosomes 1, 5, and 19, as they share almost identical consensus sequences at their respective loci where the centromere is formed (Figure 6). This allowed us to ask whether or not the centromeric repeats of each of these chromosomes share more DVNs than do those of non-centromeric repeats. For this purpose, we aligned in a single ClustalW analysis the sequenced repeats of the three chromosomes, including the two sets belonging to the two chromosome 5 homologues of this study, as they are evolutionarily independent. The centromere-associated subsets clearly resemble one another more than the other repeats do, as they are much more intermingled. This can be seen in the representation shown in Figure 12, in which the subsets of the CENP-A associated repeats (top) belonging to the four chromosomes are closer to each other in terms of diagnostic nucleotide variations than are those of their pericentromeric counterparts (bottom). We investigated whether this property was still observed when the DVNs shared by at least 5% of the repeats were discarded because of their larger contribution to the ClustalW alignment. This was still the case, possibly indicating that a greater degree of selection is acting on the centromere-associated repeats in comparison to the pericentromeric repeats, which would be freer to diverge.

The same did not hold true, however, when similar comparisons were made between E1-6 and IPE1-6 and between similar subsets of GM 10321 and GM 10498 from chromosome 17 (not shown). It is noteworthy, however, that both centromeric and pericentromeric alphoid repeats of chromosomes 17 and 21 have not yet reached the degree of divergence observed for chromosomes 1, 5, and 19 (see Discussion).

CENP-A is strongly associated with alpha satellite repeats containing the CENP-B box [19]. Indeed, the alpha satellite arrays analysed here all exhibit such boxes. It was interesting to examine how the CENP-B nucleotides, which are essential for the binding of CENP-B proteins [20], were affected by mutations and by their spreading to other repeats. In most cases, they were either completely unaffected or only rarely so, with the exception of those of chromosomes 1, 5, and 19, especially in the repeats associated with CENP-A (not shown).

## Discussion and Conclusion

We have previously suggested that alphoid arrays made of highly homogeneous HORs evolve by homogenisation/amplification runs which differentiate them into a series of domains that bear almost identical haplotypes as defined by Diagnostic Variant Nucleotides [6]. Moreover, we showed that exchanges between homologues are essentially absent, with each homologue evolving within its particular lineage through the accumulation of unequal crossovers during germ line mitosis. Conversion was viewed as primarily introducing divergence between the repeats.

In the present paper, we have revisited and extended these observations through the analysis of additional chromosomes (1, 3, 5, 19, and 21). We have also examined how the repeats corresponding to the CENP-A nucleosomes of the centromere behave with respect to these evolutionary mechanisms.

### The D1Z5 locus is archetypical of the evolution of highly homogeneous alphoid arrays

We first examined the long stretch of alpha satellite DNA from locus D1Z5 (1q), which was available in databases. Knowing the map position of each repeat allowed us to confirm that the same observations could be made along its 55 homogeneous 1866 bp-long tandem repeats, albeit with greater complexity than what was observed and predicted in our previous paper [6]. It is composed of two superimposed domains with relatively short subdomains, showing that the process of homogenisation/amplification acts at a high frequency and provides a somewhat constant flux through the generations. Exchanges were almost absent between the two domains due to the presence of an impassable barrier separating them that was generated by the duplication of a relatively divergent repeat by unequal crossing over, thereby increasing the distance between the most proximal repeats.

When six chromosome 21 homologues were examined, we were able to confirm that the number of DVNs was quite small. In contrast to our previous report, however, only a fraction of them was shared between the homologues, indicating that each chromosome 21 lineage "chooses" its DVNs to be homogenised/amplified independently from the others. We cannot, however, conclude that this "choice" is totally random (see below). A large minority of the copies shared the same haplotype, indicating that the D21Z1 alpha satellite repeats have been relatively stable over time, or, alternatively and more likely, that the formation and fixation of this locus occurred relatively recently.

Loci D1Z7 and D19Z3 from chromosomes 1 and 19 exhibited ClustalW alignment patterns that were similar to that of BAC BX248407 (D1Z5), with an even larger number of DVNs. They are also comprised of domains and subdomains superimposed on one another and exhibit obvious conversion events. In the absence of a position map for the analysed repeats, however, it was difficult to determine whether the pairs of relatively diverged copies that are observable constitute, as with D1Z5, barriers between different domains. When the two chromosome 5 homologues were compared, the number of DVNs was much lower, although it was still larger than that of the D21Z1 locus. Their DVN distributions were largely different, again showing independent homogenisation/amplification runs in the two corresponding lineages. They represent intermediate states of nucleotide variation and exchange between chromosome 21 and chromosomes 1 and 19.

An important property related to the molecular evolution of highly homogeneous alphoid arrays emerges from these analyses: all the chromosomes analysed to date are subjected to a constant flux of exchanges occurring during the series of mitoses in the germ line. This phenomenon probably takes place in each generation and is apparently an intrinsic property of the tandemly arranged highly homogeneous alphoid HORs. Given the differences in the extent of the phenomenon on different chromosomes, it is difficult to say if it depends on the particular chromosome involved or, more likely, on the amount of time that has elapsed since the formation of the homogeneous alphoid array.

The existence of several alphoid arrays coexisting within the centromeric regions of a number of chromosomes might be a consequence of this continuous process: with time, the divergence between the repeats has become so high in certain arrays that they are no longer capable of forming a centromere. Beyond a certain level of divergence, the process of accumulation of unequal crossovers stops and they drift, ultimately becoming monomeric. This model fits well with the observation made by Schueler et al [21] that the monomeric alphoid arrays present on Xp are ancestral to the highly homogeneous block where the centromere is formed [22]. The same is true of chromosome 17 [23].

### Which status for the repeats associated with CENP-A?

We wanted to investigate the evolutionary behaviour of the minority of repeats that are engaged in the actual centromere. Alphoid homogeneous arrays can be very small, as on chromosome 21 where the array can be less than 100 kb long [24]. It was not surprising, therefore, that on chromosomes 1, 5, and 19, almost no repeats representative of those associated with CENP-A were detected in the bulk set of repeats. This confirmed that the proportion of alphoid repeats from a homogeneous array that is engaged in the real centromere can be very low. The overall features of these repeats were shown, however, to be similar to those exhibited by the bulk repeats. They are therefore evolving in the same way.

The sizes of the domains and subdomains they exhibit could not be estimated at present, but if they are similar to those that are supposed to exist within the pericentromeric alphoid repeats such as within BX248407, they would be compatible with the interspersed structure of human CENP-A and histone H3 nucleosomes [16,17]. The most striking feature of this analysis is that the DVNs that the CENP-A associated repeats have "chosen" for homogenisation/amplification are quite distinct from the other repeats. These small sequence differences might reflect a certain degree of sequence dependence for the recruitment of the proteins that constitute the CENP-A centromeric nucleosome-associated complex [8]. At the same time, when several homologues were examined the DVNs exhibited by these repeats (here chromosomes 21 and 5) were largely different, consistent with an absence of a strict sequence dependence for CENP-A to bind directly to alpha satellites, as reported by Conde e Silva et al [18].

A more plausible explanation for this difference in DVNs could be that during the constant process of change that supposedly leads to the loss of the capacity of the alphoid repeats to form an active centromere, certain nucleotide changes do not spread at the same rate within the CENP-A associated repeats. Alternatively, during the proposed centromere meiotic drive [25,26], some haplotypes could be actively selected against to preserve the centromere integrity of the unique remaining cell that is available for fertilization during female meiosis II.

The comparison carried out between chromosomes 1, 5, and 19, which share almost identical consensus sequences at their respective centromeric loci, supports this hypothesis. The DVNs of repeats originating from the three chromosomes were shared in higher proportions when associated with CENP-A than when recovered from the bulk. This was shown by simultaneous clustalW alignments of the repeats of the four chromosomes tested (1, 19, and two chromosome 5 homologues). We cannot, however, conclude from this analysis that the DNA sequence of the centromere-associated repeats is an important factor in its formation, even though is it possible to suggest that there are constraints upon the nucleotide variations that occur in this portion of an alphoid array.

### The CENP-A associated alphoid repeats may be found in unrelated alphoid arrays of the same chromosome

Another unexpected observation of this study was that repeats associated with CENP-A were detected on both chromosomes 1 and 19 on two unrelated but contiguous homogeneous arrays of alpha satellite DNA. This was not the case, however, for the two chromosome 5 homologues. This observation raises the possibility that centromeres can be formed by repeats originating from different alphoid arrays, provided that they are homogeneous enough. Another possibility is that there is an alternative centromere location on chromosomes 1 and 19, as has been shown in one Robertsonian fusion [27]; most fusions of this kind contain dicentric chromosomes with one of the two centromeres being inactivated. Interestingly, Sullivan and Willard [28] have described stable dicentric human X chromosomes in which the distance between the two functional centromeres is relatively small - as apparently is the case in the two chromosomes described here - thereby preventing anaphase bridge formation, chromosome breakage, and chromosome loss. It is noteworthy that in the case of the D1Z7 locus of chromosome 1, one of the two series of potential CENP-B boxes has been almost totally destroyed by mutation, whilst D1Z5 exhibits CENP-B boxes in their integrity, which could help this locus recruit CENP-A proteins [29].

### A model for the formation and maintenance of active human centromeres

With the above observations in mind, it is possible to make some suggestions and predictions concerning the formation and evolution of human centromeres at alpha satellite loci, where they are mostly found (neocentromeres are estimated to occur in approximately 0.0005%-0.0014% of live births [30]).

It has been previously pointed out that the alphoid repeats that are capable of contributing to an active centromere must be part of an extremely homogeneous higher-order multimeric repeat unit array that is uninterrupted by retrotransposons [31,6]. They are submitted to continuous nucleotide changes which spread at high rates to adjacent repeats. This constitutes a progressive process that probably depends on the amount of time that has elapsed since the homogeneous array was formed. This fits well with the differences found between chromosome 21 on the one hand and chromosomes 1 and 19 on the other, with chromosome 5 being intermediate between them with respect to both the number of detected DVNs and the proportion of sporadic mutations.

When a highly homogeneous array has been created, a functional centromere can be formed. This is clearly possible with a large variety of alpha satellite DNA sequences, since most chromosomes exhibit largely divergent ones. The intrinsic ability of highly homogeneous multimeric tandem repeats to homogenise/amplify by accumulating unequal crossovers continues to act upon repeats that are almost identical. This identity is slowly undermined by the accumulation of random mutations, but as long as domains compatible with the formation of an active centromere exist, the array continues to play its functional role. In this study, this is the case with D21Z1 and D5Z2, which have not yet accumulated enough divergence to affect this compatibility, in contrast to chromosomes 1 and 19, in which CENP-A associated higher-order alphoid repeat units have been recovered in a second homogeneous alphoid array.

We do not know, however, if these repeats are part of the active centromere or if they are part of a potential alternative centromere that is in the process of being formed. This might represent a general way of ensuring the stability of human chromosomes over time, as an alternative to the exceptional possibility of being rescued through neocentromere formation. Significantly, five chromosomes with neocentromeres have been described in which the alphoid array within which the centromere is normally formed is still present, three on chromosome Y, one on chromosome 3, and one on chromosome 4 [30]. It is interesting to note that there is apparently only one alphoid array in each of these three chromosomes, meaning that there is no possibility for a centromere to form within another array if the unique one loses its capacity to bind the CENP-A centromeric nucleosome associated complex. The number of neocentromere-containing chromosomes reported to date could be largely underestimated because they are not associated with clinical defects, in contrast to those in which the alphoid sequences have been lost [30]. The defects of the old inactivated centromeres have not been characterized, although it has been suggested that there might have been a partial deletion of the alphoid DNA, which seems unlikely if one refers to the extreme variations of alpha satellite DNA found in normal chromosomes [24]. We rather think that the normal destiny of a centromere is to be lost over time and to be replaced by a new one, most often within the same alphoid array or in a second one, with neocentromeres of the above type representing in this case a transient possible way to rescue a chromosome with an impaired centromere [6].

## Methods

### Cell Culture and DNA samples

Cells were grown in RPMI 1640 supplemented with 10% foetal bovine serum (Gibco) and penicillin-streptomycin (100 U/ml) in 95% air/5% CO_2 _atmosphere at 37°C. Several hybrid cell lines were used in this study, either as sources of DNA or for immunoprecipitation of CENP-A associated chromatin: six contained a single chromosome 21 each; they were a generous gift from Dr Stephanie L. Sherman (Emory University Medical School, Dept of Human Genetics). These cell lines had been generated from two trisomic 21 probands [32] and had been previously genotyped to ensure that they corresponded to the two chromosomes 21 of maternal origin and one of paternal origin. Hybrid cell lines containing one chromosome 1 (GM 13139), one chromosome 19 (GM 10449), and two lines with one chromosome 5 each (GM 10114 and GM11714) were also used. Two hybrid cell lines with one chromosome 17 each, GM10321 and GM10498, were also used. All were purchased from Coriell Cell Repositories.

Other DNA samples originating from hybrid cell lines, containing one normal chromosome 3 either as the unique human constituent (GM 10253) or accompanied by others (HY.46BF (X, 6, 8, 13), HY95A1T4 (X, 5, 7f, 8f, 10, 11, 14)), were provided by Dr M Rocchi (University of Bari). To analyse the alpha satellite DNA sequences of the unique normal chromosome 3 they contained, several pairs of oligonucleotide primers were tested in varying PCR conditions to ensure that it was possible to recover the alpha satellite sequences of chromosome 3 without contamination by those of other chromosomes (not shown).

### Chromatin Immunoprecipitation (ChIP)

Cells were treated with 1% formaldehyde for 10 min at room temperature to form DNA protein cross-links. They were then collected by centrifugation at 1,000 rpm for 5 min at 4°C and resuspended in a Swelling Buffer (0.1 M Tris-HCl at pH 7.6, 10 mM KOAc, 15 mM MgOAc, Roche Protease Inhibitors Mix) for 20 min on ice. Cells were then collected by centrifugation, resuspended in a Nuclei Lysis Buffer (50 mM Tris-HCl at pH 8.0, 10 mM EDTA pH 8.0, 1% SDS, Roche Protease Inhibitors Mix), and incubated on ice for 10 min. Homogenisation with a Dounce homogenizer (15 strokes on ice) was then performed and the lysates were sonicated (Diagenode Bioruptor Sonicator). Samples were pre-cleared with PMSF (1 mM) and Protein A/G PLUS-Agarose beads (Santa Cruz), and incubated on ice for 15 min. Samples were then centrifuged at 4,000 rpm for 5 min at 4°C, and the supernatant was incubated with anti-CENP-A antibodies overnight at 4°C on a rotating platform. Anti-CENP-A antibodies were either a generous gift from Dr A Choo (University of Melbourne) or purchased from Covalab, with similar results. To collect the immunoprecipitated complexes, Protein A/G PLUS-Agarose beads were added and incubated at 4°C for 1 h 30 min on a rotating platform. The beads were then recovered by centrifugation (at 4°C for 5 min at 4000 rpm) and washed once with 1 ml of RIPA Buffer (150 mM NaCl, 50 mM Tris-HCl at pH 8, 0.1% SDS, 0.5% sodium deoxycholate, 1% NP40), once with 1 ml of HI Salt Buffer (0.5 M NaCl, 50 mM Tris-HCl at pH 8.0, 0.1% SDS, 1% NP40), once with 1 ml of LiCl Buffer (0.25 M LiCl, 50 mM Tris-HCl at pH 8.0, 0.5% sodium deoxycholate, 1% NP40) and twice with 1 ml of TE Buffer (10 mM Tris-HCl at pH 8.0, 1 mM EDTA). Each washing step was performed for 10 min at 4°C on a rotating platform and the sample was then centrifuged at 4,000 rpm for 5 min at 4°C. Bound immunocomplexes were then incubated twice with 200 μl of Elution Buffer (2% SDS, 0.1 M NaHCO_3_, 10 mM DTT) for 15 min at room temperature on a rotating platform. After centrifugation at 4,000 rpm for 5 min at room temperature, the two supernatant fractions were then recovered, pooled, and the DNA protein cross-links reversed with 5 M NaCl overnight at 65°C. To recover the DNA, samples were mixed with 0.5 M EDTA, RNase for 30 min at 37°C. 1 M Tris-HCl at pH 7.6 and Proteinase K were then added and the samples incubated at 45°C for 2 hours. DNA was then recovered by phenol-chloroform extraction and ethanol precipitation and resuspended in 40 μl H_2_O.

In a number of species, including man, anti-CENP-A antibodies bind with high specificity to the loci where centromeres are formed, either within the satellite DNA arrays which contain them or within the neocentromeres which substitute for them in a limited number of cases. The control in this study was therefore *de facto *included when the repeats of the alphoid array as a whole were compared to those associated with CENP-A. The high specificity of anti-CENP-A antibodies is also exemplified by experiments in which DNA sequences were recovered, either by ChIP or by ChIP on chip, from the very same genomic sites where neocentromeres had been shown previously to be formed [33,34]. Finally, in the ChIP experiments performed in this study with the two independent hybrid chromosome 5 cell lines, using the very same immunoprecipitates, CENP-A associated repeats could be recovered only from one of the two alphoid arrays present in their centromeric regions (see Figure 11).

### PCR, cloning and DNA sequencing

DNA samples were PCR amplified using Promega *GoTaq *Flexi DNA polymerase and associated buffer. The annealing temperature was 55°C. PCR products were resolved by electrophoresis on 1% agarose gels and the DNA fragments of interest purified using the QIAGEN QIaquick Gel Extraction Kit. Cloning was performed with the Promega pGEM-T Easy Vector System. Positive individual clones were recovered and grown in 96-well plates. 17-3A: (5'-TTATGGTCACATAAAAACTG-3') and 17-4A: (5'-ATCTACTTGCAGTTTCTACAG-3') were primers for chromosome 17; 13/21-3A (5'-CTTCTGTCTAGATTTTAGA-3') and 13/21-1B (5'-CATAGAGATGAACATGG-3') for chromosome 21; 3A (5'-TCTGCAAGTGGATATTTAAA-3') and 3B (5'-TGAGTTGAACACACACGTAC-3') for chromosome 3; 1A (5'-TTTCAACCTGAACTCACAAG-3') and 1B (5'-CTCATCAAAGCTACATGGAA-3') for D1Z5; D5Z2-A (5'-ATTCAACTCACAGAGTTGAACGA-3') and D5Z2-B (5'-GAATGTACACAACACAAGGAAGC-3') for alpha satellite arrays shared by chromosomes 1 (D1Z7), 5 (D5Z2), and 19 (D19Z3). DNA sequencing was performed by Cogenics. DNA sequences were analysed using the clustalW alignment program [35].

### FISH analysis

Prometaphase spreads were prepared from PHA-stimulated human peripheral blood lymphocytes cultured for 72 hours and with BrdU treatment. BAC RP11-483B6 DNA was labelled with biotin-16-dUTP (Roche Diagnostics, France) and plasmid pZ5.1 DNA with digoxigenin-11-dUTP (Roche Diagnostics, France) by nick translation using a commercial kit (Roche Diagnostics, France). Probes were suspended in 60% dextran sulphate/formamide/SSC hybridization buffer. Before FISH, slides were treated with pepsin. Human Cot1 DNA (Roche Diagnostics, France) was added as a competitor for the BAC RP11-453B6 probe. Slides denaturation was performed in a 70% formamide, 2× SSC solution, pH 7 at 73°C for 3 minutes and probes were denatured in a waterbath at 73°C for 5 minutes. Slides and probes were incubated overnight in a moist chamber at 37°C for hybridization. Posthybridization treatment included two 10 minutes washes in 50% formamide, 2× SSC pH 7 followed by one 7 minutes wash in 2× SSC pH 7 and one 7 minutes wash in 2× SSC, 0.1% NP-40 at 46°C. Subsequent cytochemical detection of the hybridization signals was performed with streptavidin-Alexa 594 (Invitrogen SARL, France) and antidigoxigenin-fluorescein (Roche Diagnostics, France). Chromosomes and nuclei were counterstained with DAPI-II (Abbott Vysis, France). The signal was visualized by digital imaging microscopy (Leica Leitz DM RB, Germany) using a cooled charge-coupled device camera (MetaSystems, Germany). Image capture was performed using Isis software (MetaSystems, Germany).

## Authors' contributions

NP performed the experimental work, except for FISH experiments which were performed by JP. GR and NP analysed data. GR wrote the paper which was read, discussed and approved in its final version by all authors.

## Supplementary Material

Additional file 1 **ClustalW alignment of repeats from alphoid array D21Z1 of chromosome 21: E4-E6**. The DVNs are shown in the upper line, with their positions along the 1866 bp long HOR. A minority of them are shared by the six homologues. No DVNs at all were exhibited by 15 (E4), 13 (E5), and 16 (E6) repeats; they might, however, differ by a few sporadic mutations (less than 0.2% on average). Click here for file

Additional file 2 **ClustalW alignment of the sequenced repeats of D19Z3 from hybrid cell line GM 10449**. This was performed with all sequenced repeats, including those in which a DVN was only shared by two repeats. Those DVN positions are not included here for more clarity. Click here for file

Additional file 3 **ClustalW alignment of the IPE4-IPE6 CENP-A associated repeats recovered by immunoprecipitation from chromosome 21**. The minority of DVNs that are shared between most of the six homologues are shown in the upper line, with their positions along the 1866 bp long HOR indicated. No DVNs at all were exhibited by 13 (IPE4), 19 (IPE5), and 29 (IPE6) repeats; they might, however, differ by a few sporadic mutations (less than 0.2% on average) Click here for file

Additional file 4 **ClustalW alignment of the CENP-A associated repeats recovered by immunoprecipitation from chromosome 1 (IP Chr1)**. Above each set of sequences, the nucleotide positions where a significant subset of repeats share the same DVNs are indicated. ClustalW alignment was performed with all sequenced repeats, including those in which a DVN was only shared by two repeats. These DVN positions are not included here for more clarity. Click here for file

Additional file 5 **ClustalW alignment of the CENP-A associated repeats recovered by immunoprecipitation from chromosome 19 (IP Chr19)**. Above each set of sequences, the nucleotide positions where a significant subset of repeats share the same DVNs are indicated. ClustalW alignment was performed with all sequenced repeats, including those in which a DVN was only shared by two repeats. Those DVN positions are not included here for more clarity. Click here for file
