# Supplementary figures and images for: Molecular and evolutionary characteristics of the fraction of human alpha satellite DNA associated with CENP-A at the centromeres of chromosomes 1, 5, 19, and 21
Source: BMC Genomics. 2010 Mar 23;11:195. doi: 10.1186/1471-2164-11-195 (PMC2853522; doi:10.1186/1471-2164-11-195)

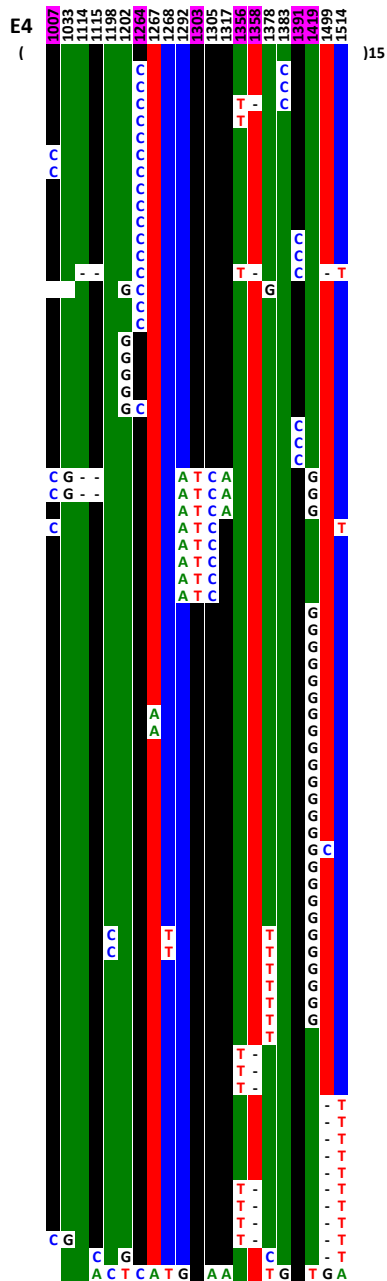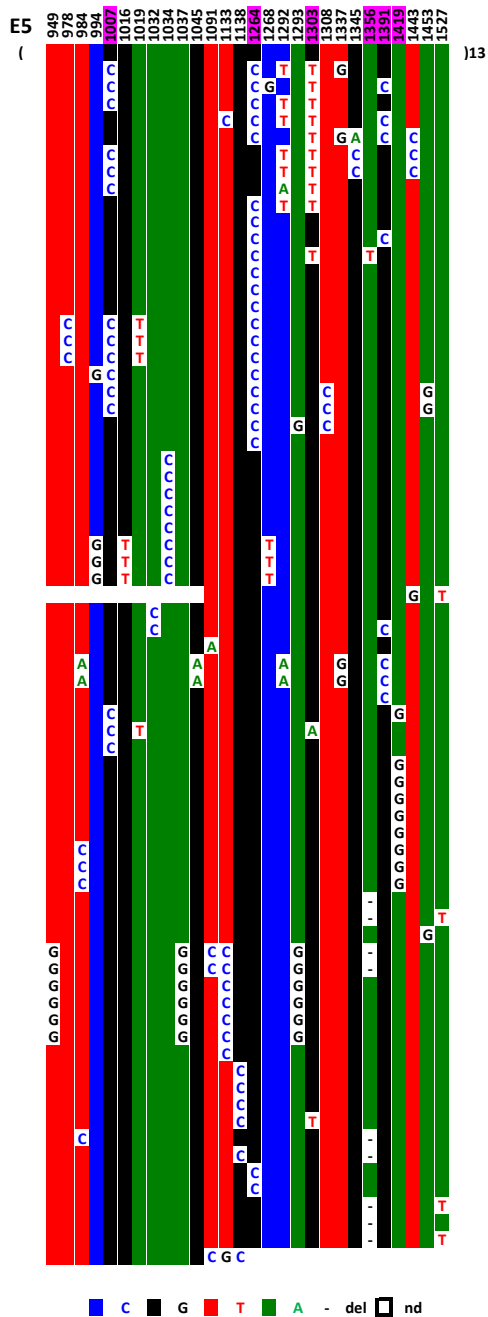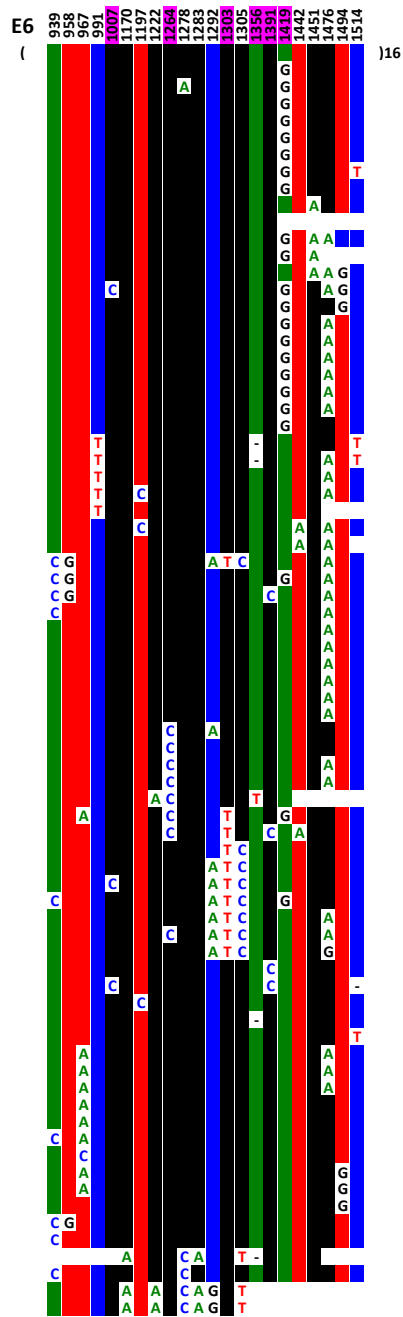

Supplement: Additional file 1 — ClustalW alignment of repeats from alphoid array D21Z1 of chromosome 21: E4-E6. The DVNs are shown in the upper line, with their positions along the 1866 bp long HOR. A minority of them are shared by the six homologues. No DVNs at all were exhibited by 15 (E4), 13 (E5), and 16 (E6) repeats; they might, however, differ by a few sporadic mutations (less than 0.2% on average). [file 1471-2164-11-195-S1.PDF]

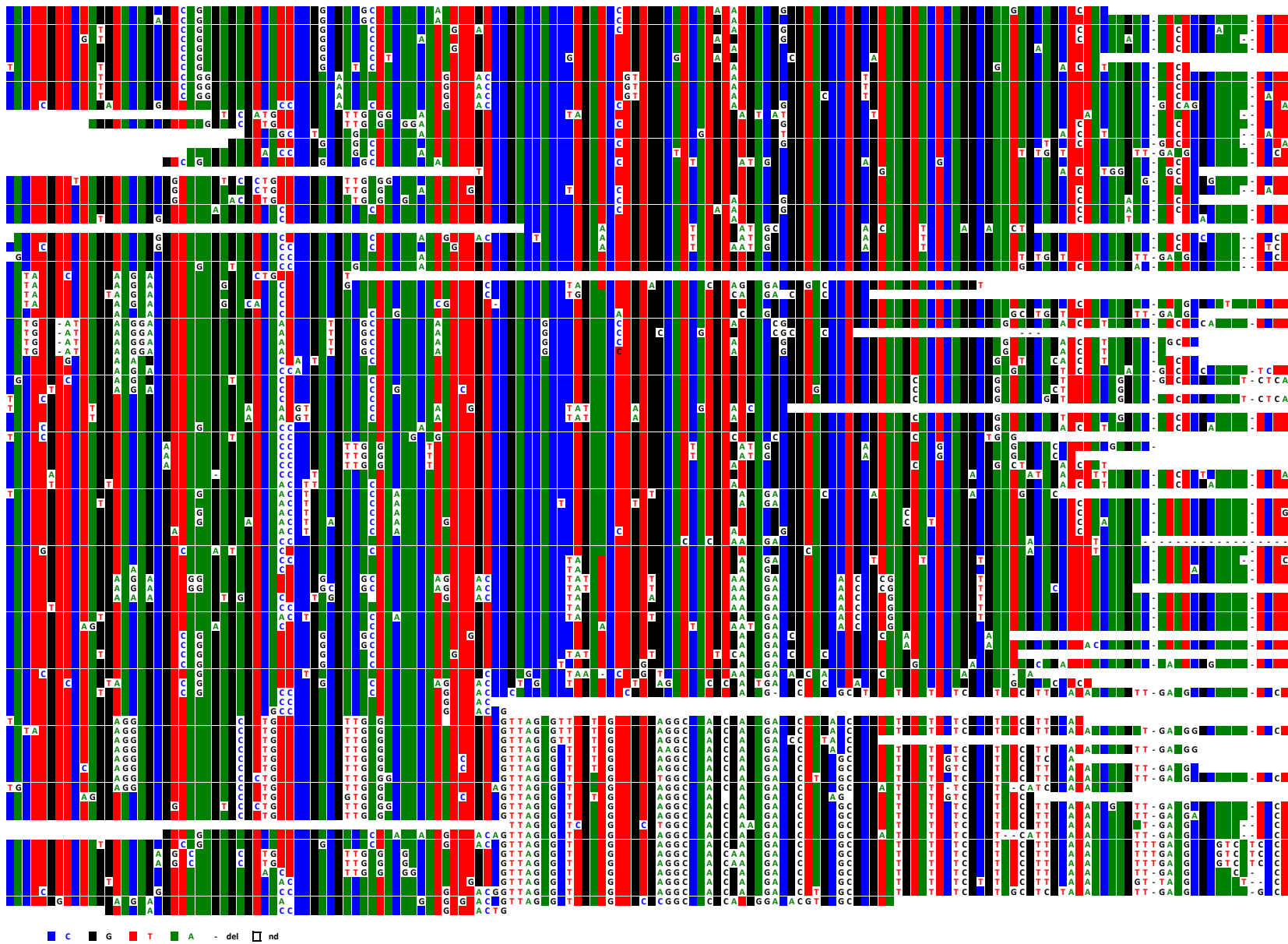

Supplement: Additional file 2 — ClustalW alignment of the sequenced repeats of D19Z3 from hybrid cell line GM 10449. This was performed with all sequenced repeats, including those in which a DVN was only shared by two repeats. Those DVN positions are not included here for more clarity. [file 1471-2164-11-195-S2.PDF]

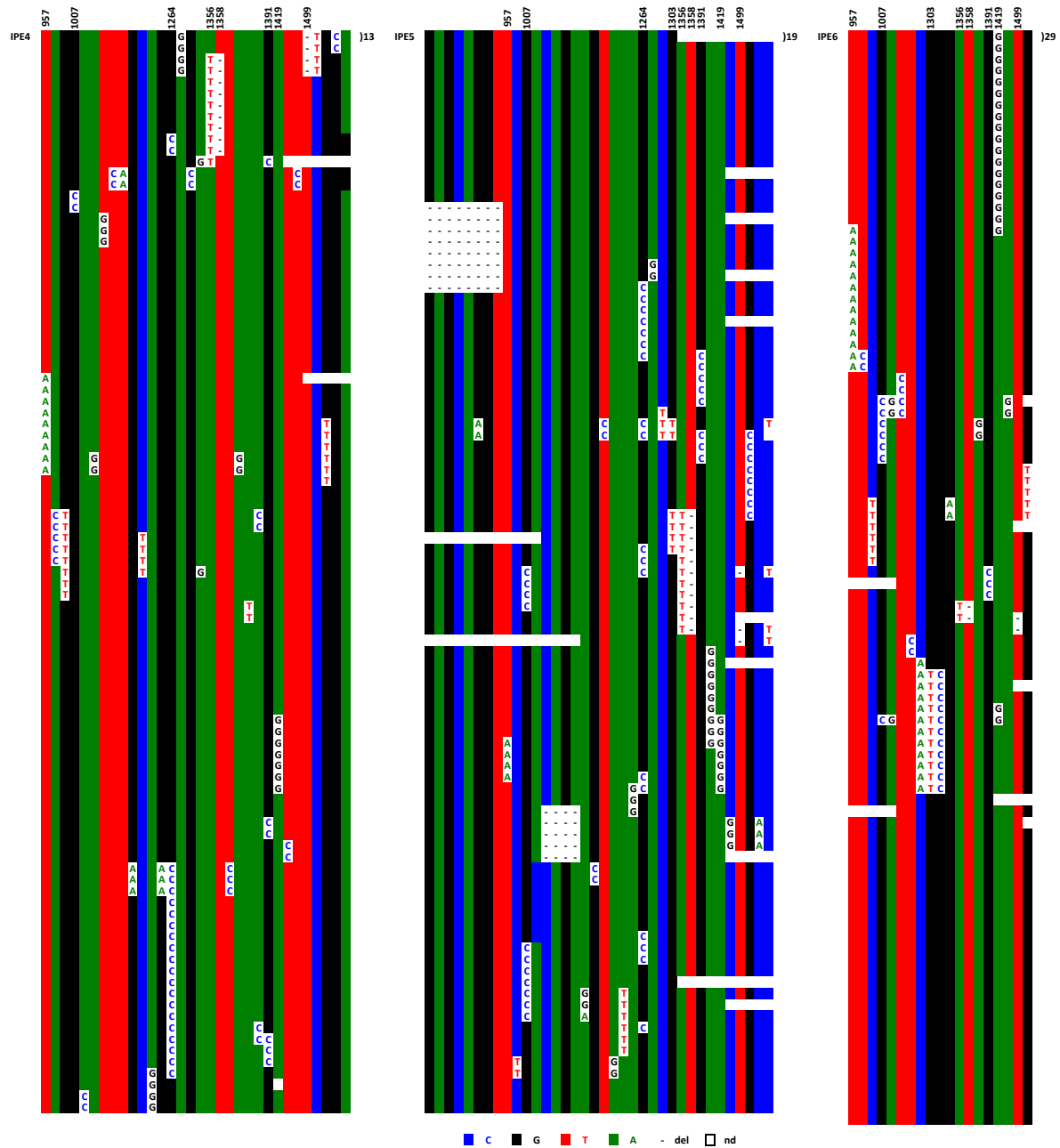

Supplement: Additional file 3 — ClustalW alignment of the IPE4-IPE6 CENP-A associated repeats recovered by immunoprecipitation from chromosome 21. The minority of DVNs that are shared between most of the six homologues are shown in the upper line, with their positions along the 1866 bp long HOR indicated. No DVNs at all were exhibited by 13 (IPE4), 19 (IPE5), and 29 (IPE6) repeats; they might, however, differ by a few sporadic mutations (less than 0.2% on average) [file 1471-2164-11-195-S3.PDF]

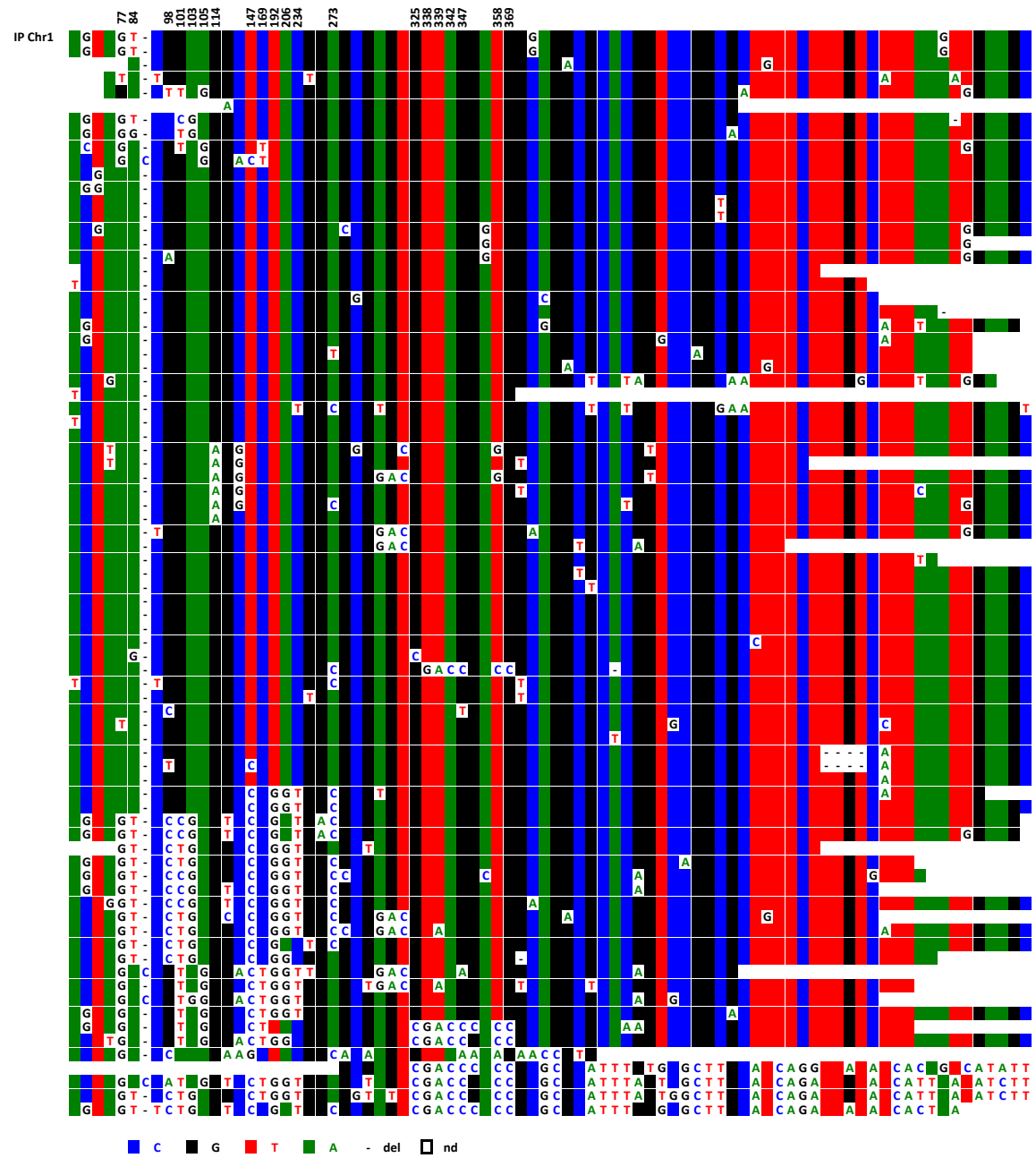

Supplement: Additional file 4 — ClustalW alignment of the CENP-A associated repeats recovered by immunoprecipitation from chromosome 1 (IP Chr1). Above each set of sequences, the nucleotide positions where a significant subset of repeats share the same DVNs are indicated. ClustalW alignment was performed with all sequenced repeats, including those in which a DVN was only shared by two repeats. These DVN positions are not included here for more clarity. [file 1471-2164-11-195-S4.PDF]

IP Chr19

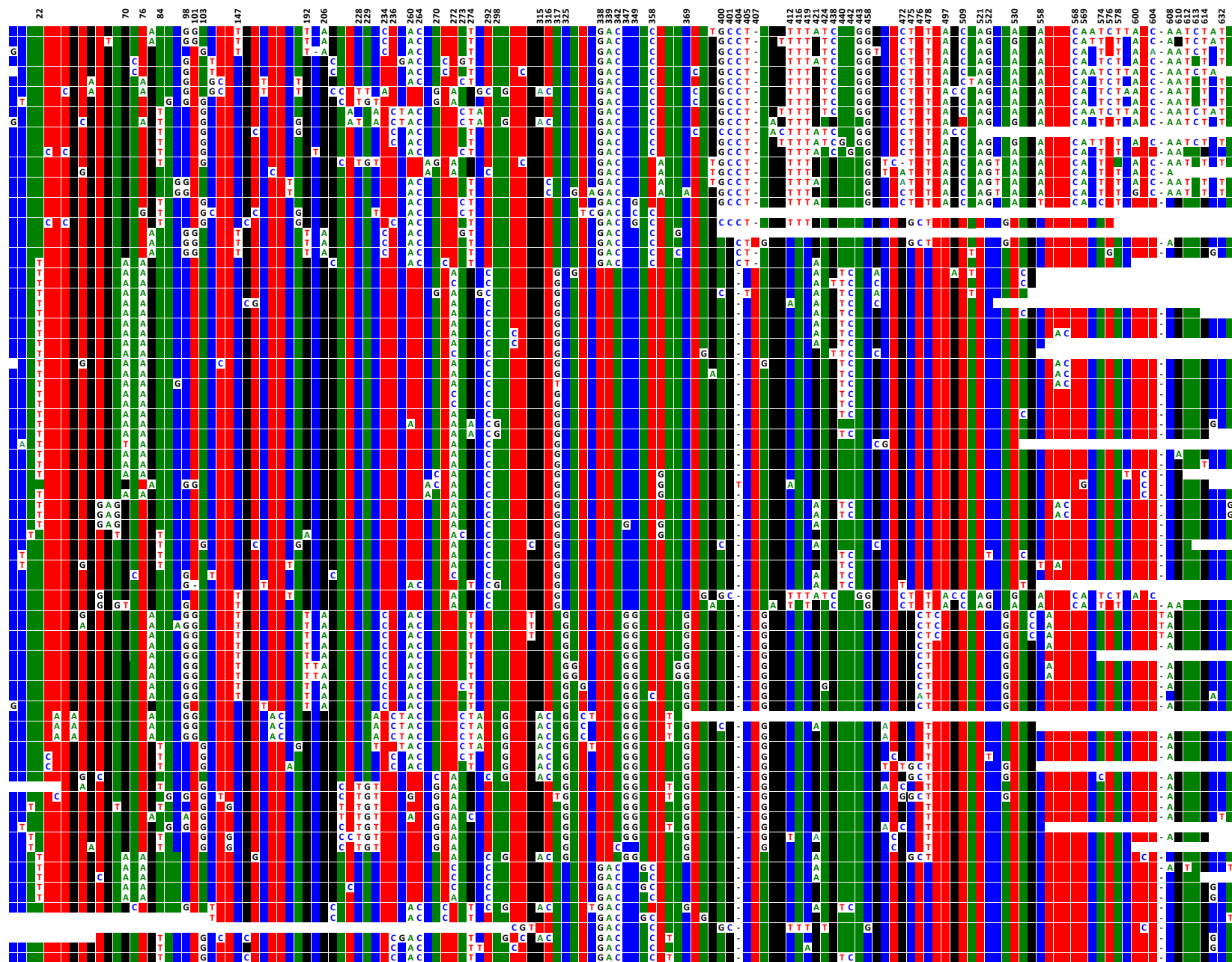

Supplement: Additional file 5 — ClustalW alignment of the CENP-A associated repeats recovered by immunoprecipitation from chromosome 19 (IP Chr19). Above each set of sequences, the nucleotide positions where a significant subset of repeats share the same DVNs are indicated. ClustalW alignment was performed with all sequenced repeats, including those in which a DVN was only shared by two repeats. Those DVN positions are not included here for more clarity. [file 1471-2164-11-195-S5.PDF]
